# Estradiol and progesterone regulate proliferation and apoptosis in colon cancer

**DOI:** 10.1530/EC-18-0374

**Published:** 2019-02-04

**Authors:** Corina Verónica Sasso, Flavia Eliana Santiano, Fiorella Campo Verde Arboccó, Leila Esther Zyla, Silvana Noemí Semino, Martin Eduardo Guerrero-Gimenez, Virginia Pistone Creydt, Constanza Matilde López Fontana, Rubén Walter Carón

**Affiliations:** 1 Instituto de Medicina y Biología Experimental de Cuyo (IMBECU), CCT-Mendoza CONICET, Mendoza, Argentina; 2 Universidad de Mendoza, Mendoza, Argentina; 3 Hospital Universitario, Universidad Nacional de Cuyo, Mendoza, Argentina

**Keywords:** colon cancer, 17-beta estradiol, progesterone, apoptosis

## Abstract

Epidemiological studies describe estrogens as protectors in the development of colon cancer in postmenopausal women treated with hormone replacement therapy. However, the role of progesterone in colon cancer has been minimally studied and the results are controversial. For the above, the objective of this work was to determine the hormonal regulation exerted by natural ovarian steroids on proliferation and apoptosis in an experimental model of colon cancer in ovariectomized rats treated with 17-beta estradiol and progesterone. Sprague–Dawley rats were exposed to the carcinogen 1,2-dimethylhydrazine to induce colon tumors. Thirty days later, the rats were ovariectomized and treated with estradiol (60 μg/kg), progesterone (10 mg/kg), estradiol plus progesterone (60 μg/kg and 10 mg/kg) or vehicle. We observed no significant differences in colon cancer incidence and tumor multiplicity between the groups. Nevertheless, we observed a decrease in PCNA expression and a greater number of apoptotic index, higher expression of caspase 3, cleaved PARP and cleaved caspase 8 in tumors, confirming the activation of the extrinsic pathway of apoptosis by the combined treatment. In addition, we observed a higher expression of estrogen receptor beta in these tumors. We conclude that the action of both hormones, estradiol and progesterone, is necessary to reduce proliferation and increase apoptosis in colon tumors, probably through estrogen receptor beta activation.

## Introduction

Colorectal cancer (CRC) is the third most common cancer and one of the tumors with the highest incidence and mortality worldwide, with an increasing projection for the coming decades. CRC incidence and mortality rates are 30 and 40%, respectively, higher in men than in women (1). Since the Women’s Health Initiative in 1991 (2), and several epidemiological studies, the ovarian steroids were considered protectors against the development of CRC. Different studies in animal models showed a lower risk of CRC in the presence of estrogens (3, 4). Nevertheless, some studies indicate that, once the disease has developed, estrogens inhibit cell proliferation, while others suggest they induce mitogenic effects (5). The oncogenic effects of estrogens have been widely studied in breast and ovarian cancer, but little is known about its action in colon cancer (6). Regarding their receptors, it is known that estrogen receptor beta (ERB) is the predominant isoform in the colon (7, 8, 9) and that its expression is lost during the progression of colon cancer, suggesting that it would play an important role in the progression of this disease (10, 11). Recent studies involving tumor samples from patients with CRC have shown that elevated expression of ERB is associated with a better prognosis, supporting its role as a possible target for chemoprevention (12, 13). Experiments carried out on ERB-knockout mice conclude that its loss leads to an increase in proliferation, loss of differentiation and decrease in apoptosis in the colon epithelium, suggesting an important role of this receptor in the normal organization and structural maintenance of the colon (11). Moreover, colon cancer cell lines have been reported to express mostly ERB after stimulation with estradiol (10–1000 nmol/L), with an induction of apoptosis dependent on the dose (6). With respect to estrogen receptor alpha (ERA), it has been reported that its expression is minimal in normal colon and in colon cancer cells (7, 14). Therefore, most of the studies demonstrate that the protective effects of estradiol in colon carcinogenesis are carried out by the ERB.

In addition to the known effects of estrogens on colon tumorigenesis, we should also consider progesterone (P4) as another of the ovarian steroids involved in this disease. There are some studies reporting the absence of the expression of progesterone receptor (PR) in colon tumors and no effect of progestins on carcinogenesis in animal models (15). However, other studies report the implication of P4. For example, the expression of PR increases in the order of normal colon-adenoma-adenocarcinoma, supporting its role on this disease (16). Furthermore, some studies propose synthetic progestins as chemopreventive agents in colon cancer (17), but little is known about the role of natural P4. The cellular effects of P4 in colonocytes have been minimally studied, and the relationship between P4 and ER is not yet elucidated (18). Thus, the objective of this work was to determine the hormonal regulation exerted by natural ovarian steroids on proliferation and apoptosis in an experimental model of colon cancer in ovariectomized rats treated with 17-beta estradiol and P4.

## Materials and methods

### Animals

Virgin Sprague–Dawley female rats were kept in a light- (lights on 06.00–20.00 h) and temperature-controlled room (22–24°C) in our animal facility. Rat chow (Cargill, Córdoba, Argentina) and tap water were available *ad libitum*.

Animal maintenance and handling were performed according to the NIH guide for the Care and Use of Laboratory Animals (NIH publication no. 86-23, revised 1991) and the UK requirements for ethics of animal experimentation (Animals Scientific Procedures, Act 1986). All the experimental procedures were approved by the Animal and Ethics Committee (CICUAL) of the School of Medicine of the National University of Cuyo, Mendoza, Argentina (0011463/2011).

### Experimental protocols

To induce colon cancer, 45-day-old rats (approximately weighting 170 g) were treated subcutaneously once a week with 1,2-dimethylhydrazine (DMH, 21 mg/kg; Sigma), for 20 weeks as previously described (19, 20). Four weeks after the first DMH dose, the rats were anesthetized by an intraperitoneal injection of ketamine-xylazine (45 and 10 mg/kg) and were ovariectomized as previously described (21). In order to study the effects of the ovarian steroids on colon carcinogenesis, they started receiving subcutaneous injections twice a week with 17-beta estradiol (E2 group, 60 μg/kg, *N* = 13; Sigma), progesterone (P4 group, 10 mg/kg, *N* = 14; Sigma), E2 and P4 (E2 + P4 group, 60 μg/kg and 10 mg/kg, respectively, *N* = 13) or vehicle (V group, vegetal oil, *N* = 10) until they were killed. All the animals were periodically controlled for symptoms, irrespective of the treatment. The same observer checked weekly the rats in the same way, looking for loss of weight, diarrhea or any sign of distress.

The incidence of colon cancer was calculated as the percentage of rats that presented tumors within the period studied. The rats were decapitated the day they were expected to receive the following hormonal dose. In consequence, they were killed 84 h after the last injection. Since the rats were killed when they exhibited symptoms of tumor presence, we compared the day of killing in order to have a parameter related to latency, and we expressed it as latency of appearance of evident symptoms. The animals without any symptom were killed at day 270 from the first DMH dose. Trunk blood samples were collected and allowed to clot at room temperature. Serum was separated and stored at −20°C until assayed for hormone determinations. Immediately after decapitation, a piece of the tumor was removed for histopathological, immunohistochemical and Western blot (WB) analysis.

### Hormone determinations

To determine the serum levels of estradiol and progesterone, the specific commercial Coat-A-Count kits (TKE21 and TKPG1; Siemens Healthcare Diagnostics Inc.) were used according to the manufacturer’s instructions. A total of 100 µL of the calibrators or 100 µL of sera were added to the precoated tubes in duplicate. One milliliter of ^125^I estradiol or ^125^I progesterone was added to each tube and incubated for 3 h at room temperature. The content of the tubes was aspirated and counted for 1 min in a gamma counter. Assay sensitivity was 8 pg/mL for estradiol and 0.02 ng/mL for progesterone. The inter- and intra-assay coefficients of variation were <10% for both hormones.

### Tumor histology

After decapitation, a small piece of tumor of each rat was fixed in 4% v/v formaldehyde for 24 h, dehydrated in ethanol and embedded in paraffin wax. Sections of 3–5 µm were cut in a HYRAX M 25 Rotary microtome (Zeiss) and stained with hematoxylin–eosin (H&E) for the analysis under the optic microscope. The tumor grade and type, the inflammation grade, fibrosis, necrosis and mitotic and apoptotic index were defined. The number of mitotic figures and apoptotic bodies present in the tumor cells in ten fields was counted under microscope at a magnification of 400×. The mitotic and apoptotic index was calculated dividing the number of mitotic figures by the number of apoptotic bodies for each tumor.

### Immunohistochemistry

Sections of 3–5 μm from each tumor underwent an antigen retrieval protocol using heat (40 min in citrate buffer 0.01 M, pH 6.0). After two washes with distilled water, the endogen peroxidase was blocked with 0.1% w/v sodium azide for 30 min. The nonspecific binding sites were blocked with 10% w/v of skim milk. The primary antibodies used were PCNA (M0879, 1:600 dilution; Dako), caspase 3 (ab4051, 1:400 dilution; Abcam), ERA (ab32063, 1:200 dilution; Abcam), ERB (ab3577, 1:750 dilution; Abcam) and PR (sc-539, 1:100 dilution; Santa Cruz Biotechnology Inc.). The antibodies were incubated overnight at 4°C in humidity chambers. A commercial kit to detect mouse and rabbit antibodies was used (Dako EnVision Systems, horseradish peroxidase, diaminobenzidine; Dako). Slides were lightly counterstained with hematoxylin to reveal nuclei, examined and photographed. The immunostaining was evaluated considering the extent, intensity and localization of immunostaining independently by two experienced researchers blinded regarding the hormone treatments, and a few conflicting scores were resolved by consensus. The intensity score was measured as follows: 0 = no staining, 1 = weak staining, 2 = moderate staining, 3 = strong staining; and a proportion score: 0 = no staining, 1 = staining less than 10% of the tumor cells, 2 = between 11 and 33%, 3 = between 34 and 65%, 4 = greater than 66%. The images were taken with a Nikon Eclipse E200 microscope (Nikon) equipped with a digital micrometrics SE High Quality camera (Accu-Scope, Commak, NY, USA) at a magnification of 400×.

### Protein isolation and WB

Total proteins in 200 mg from each tumor were isolated by mechanical homogenization with two volumes of homogenization buffer (50 mM Tris, pH 7.5, 250 mM sucrose, 10 mM benzamidine, 10 mM NaF, 5 mM sodium pyrophosphate, 20 mM glycerophosphate, 1 mM sodium orthovanadate, 1 mM PMSF, 10 mM p-nitrophenylphosphate, and aprotinin, leupeptin and pepstatin at 2 mg/L) in an ice bath. The homogenate was centrifuged at 12,500 ***g*** for 30 min and the supernatant was separated and frozen in several aliquots at −80°C until used. Proteins were quantified using the Micro BCA Protein Assay Kit (Thermo Scientific), and boiled for 5 min in loading buffer. Eighty micrograms of proteins were separated by SDS-PAGE and transferred to PVDF membranes (Immobilon-P, Merck Millipore). After rinsing and blocking with 2% w/v BSA (Sigma), the membranes were probed overnight at 4°C with antibodies targeting caspase 3 (ab4051, 1:500 dilution; Abcam), cleaved PARP (ab32064, 1:2000 dilution; Abcam), caspase 8 (ab25901, 1:1000 dilution; Abcam), ERA (ab32063, 1:2500 dilution; Abcam), ERB (ab3577, 1:3000 dilution; Abcam), PR (sc-539, 1:200 dilution; Santa Cruz Biotechnology Inc.) and B-actin (sc-47778, 1:3000 dilution; Santa Cruz Biotechnology Inc.). After a new rinsing, the membranes were probed with horseradish peroxidase-conjugated secondary antibodies anti-rabbit (sc-2004, 1:2000 dilution; Santa Cruz Biotechnology Inc.) or anti-mouse (sc-2005, 1:2000 dilution; Santa Cruz Biotechnology Inc.) for 90 min at room temperature. The membranes were rinsed and the bands were detected by chemiluminescence (ECLTM; Amersham) using a ChemiDoc XRS + System with Image Lab Software from Bio-Rad and then quantified by densitometry using digital image processing by the NIH ImageJ 1.6 freeware program. Quantitative analysis of the different protein levels was performed by determining the ratio between the specific protein and B-actin levels by densitometry.

### Expression of the *ESR1*, *ESR2* and *PGR* genes in human colon adenocarcinomas

Tumors from The Cancer Genome Atlas (TCGA) colon cancer database (https://portal.gdc.cancer.gov accessed on November 26, 2018) were evaluated. Data was programmatically downloaded using R TCGAbiolinks package. Four hundred seventy-six primary tumors were obtained and patients were classified according to their gender (males *N* = 252 or females *N* = 224). Females were further divided according to their age: greater than 50 years (*N* = 191) or less than 50 years (*N* = 33) at the time of diagnosis. Raw RNA-Seq expression counts were used and normalized using Voom transformation from R Limma package (https://genomebiology.biomedcentral.com/articles/10.1186/gb-2014-15-2-r29 accessed on November 26, 2018). Transformed gene expression distribution was depicted using boxplots and expression correlation between the three genes was evaluated using Pearson’s correlation coefficient with its corresponding *P* value. The direction of the relation was calculated using simple linear regression and depicted as a straight line with a slope in a scatterplot.

### Statistical analysis

Values are given as means ± s.e.m. of 10–14 animals per group. All statistical analyses were performed using GraphPad Prism 5.01 software (GraphPad Software Inc.). The data were analyzed by ANOVA I, with subsequent analysis of Newman–Keuls for the parametric variables, and the Kruskal–Wallis and Dunn’s for the nonparametric variables. The incidence percentages were analyzed by Fisher’s test. Differences between means were considered significant at the *P* < 0.05 level.

## Results

### The doses administered of 17-beta estradiol and progesterone reach values within the physiological range

**Figure 1:**
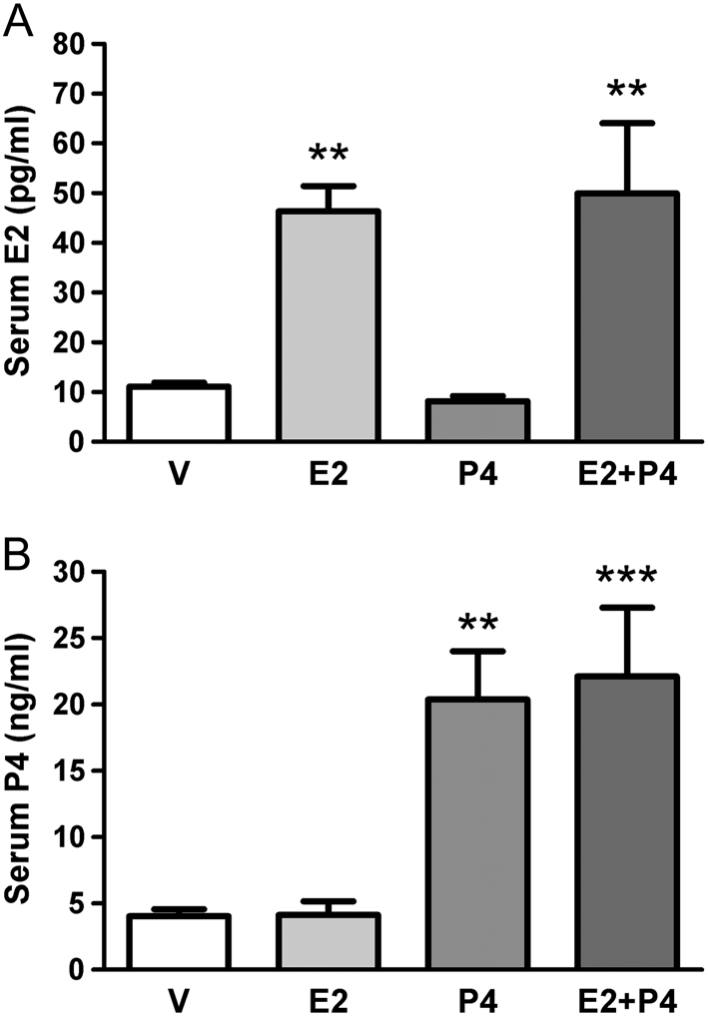
Levels of estradiol (A) and progesterone (B) in sera of Sprague–Dawley rats treated with different hormones. The rats treated with V, E2, P4 or E2 + P4 were killed at the end of the experiment and the hormone levels were determined in the sera by radioimmunoassay. **P < 0.01 and *** *P* < 0.001 compared to the other groups. The data were analyzed by ANOVA I with post analysis of Newman–Keuls.

To confirm that the dose administered of the ovarian steroids was within the normal levels, we measured the concentration of estradiol and progesterone on the sera of the rats at the end of the experiment. We observed that the group of rats treated with E2 and E2 + P4 reached the highest values of estradiol (*P* < 0.01), with an average near to 50 pg/mL (Fig. 1A). On the other hand, the levels of progesterone were higher in the groups P4 and E2 + P4, with an average of 20 ng/mL (Fig. 1B; *P* < 0.01 and *P* < 0.001). These levels of ovarian steroids are within the physiological range that has been described for Sprague–Dawley rats in the estrous cycle (23).

### Hormone treatment does not affect colon cancer incidence and tumor multiplicity, and retards the appearance of symptoms

**Figure 2:**
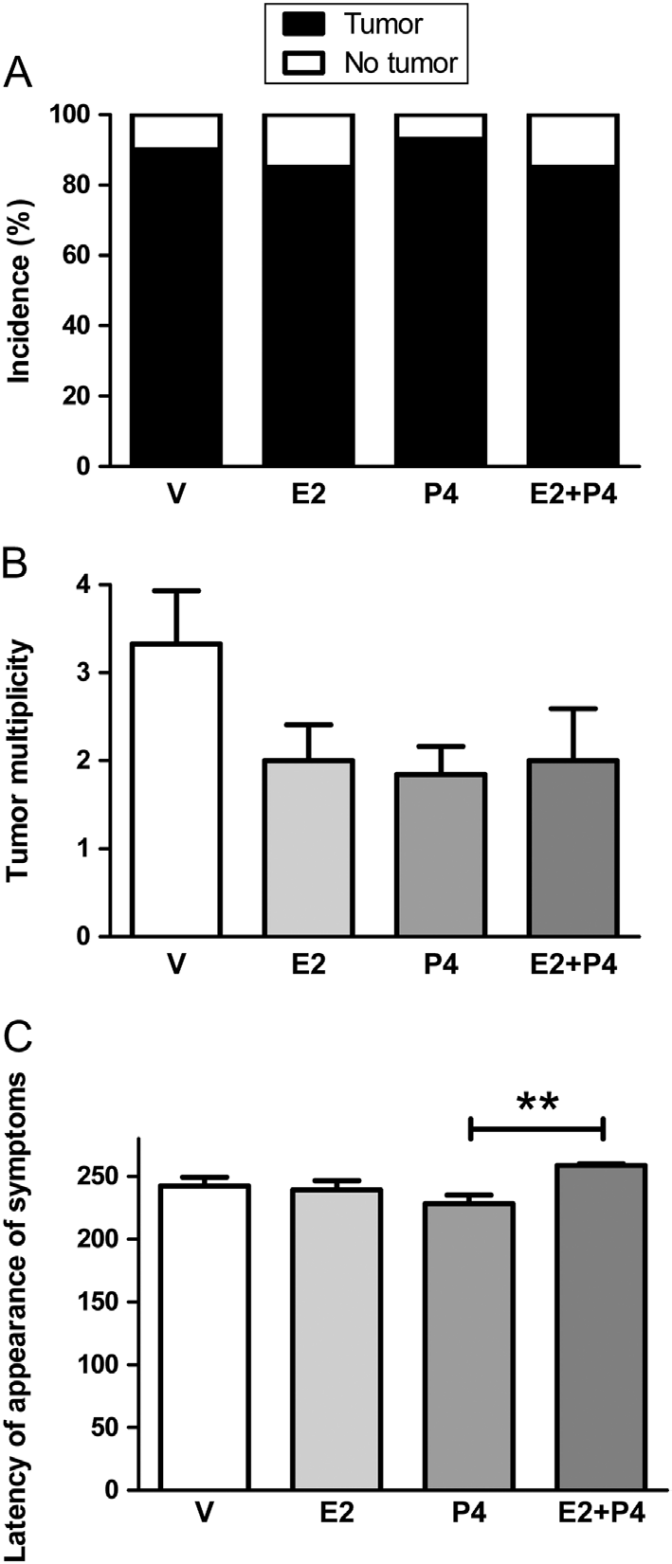
Incidence, tumor multiplicity and latency of appearance of symptoms in Sprague–Dawley rats treated with different hormones. (A) Tumor incidence. The incidence was expressed as a percentage of rats that developed colon tumors. The data were compared with Fisher’s test. (B) Tumor multiplicity. The number of tumors developed was counted for each rat and the average obtained for each experimental group was graphed. The data were analyzed by ANOVA I with subsequent Newman–Keuls analysis. (C) Latency of appearance of symptoms. The animals were killed when they presented diarrhea or weight loss, symptoms that were taken as a parameter of tumor presence. For those who did not present symptoms, the 270th day from the administration of DMH was taken as the end of the experiment. **P < 0.01 between the groups indicated with the bar. The data were analyzed by ANOVA I with subsequent analysis of Newman–Keuls.

To study the effect of ovarian steroids on the development of colon tumors, we analyzed several aspects including the tumor incidence. Rats treated with P4 or V presented an incidence of 93 and 90%, respectively (Fig. 2A). The groups treated with E2 or E2 + P4 reached an incidence of 85%. When performing the statistical analysis, no significant differences were found among these results. Also, no significant differences were observed in the number of tumors developed in each rat with regard to the different treatments (Fig. 2B).

A higher latency of appearance of evident symptoms was observed for group E2 + P4 with respect to the group treated only with P4 (Fig. 2C; *P* < 0.01), which is probably related to a higher latency in the appearance of tumors. Most of the tumors were classified as adenocarcinomas of different grades.

### **Treatment with** E2 + P4** reduces the mitotic/apoptotic index in colon tumors**

**Figure 3:**
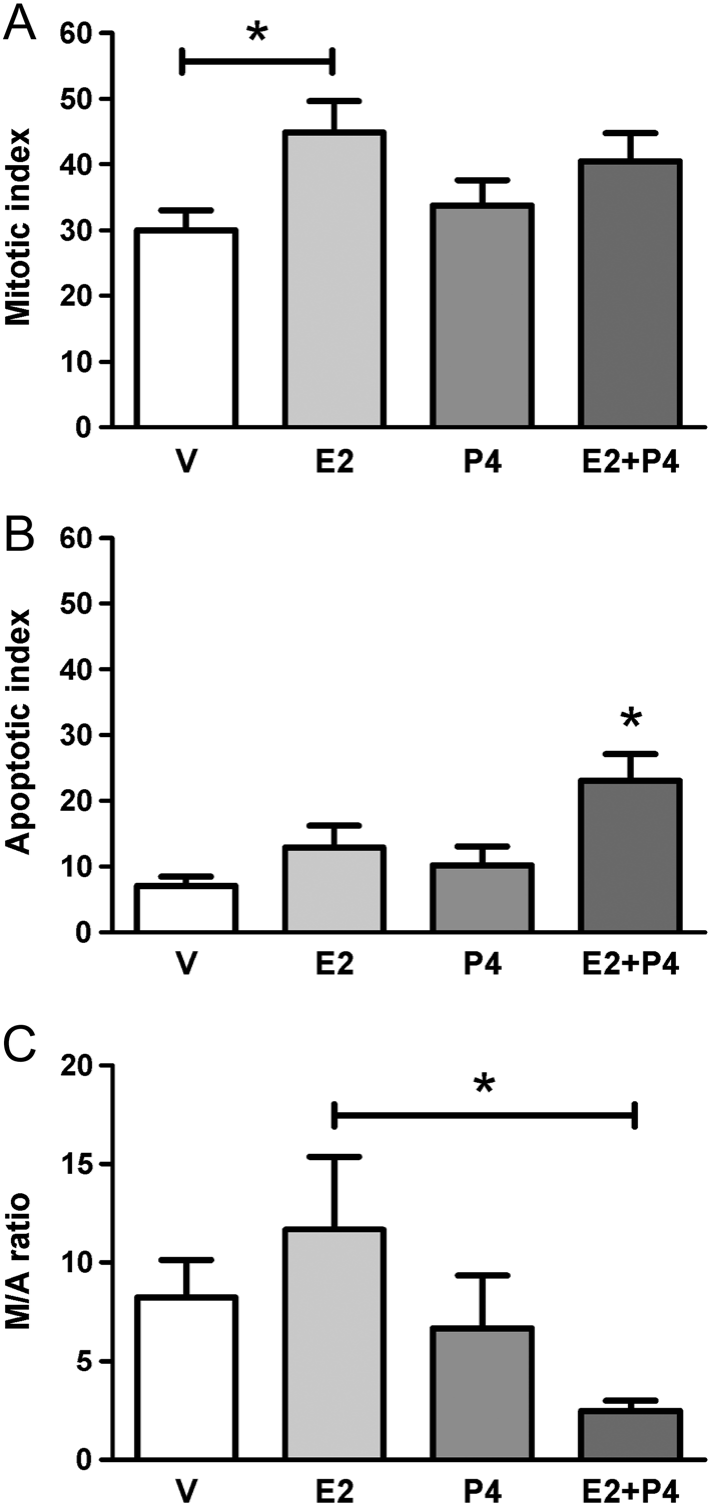
Mitotic and apoptotic indices in colon tumors from Sprague–Dawley rats. (A) The mitotic index was calculated as number of mitotic figures present in ten fields analyzed by microscopy at 400×. (B) The apoptotic index was calculated as quantity of apoptotic bodies present in ten fields analyzed by microscopy at 400×. (C) Relationship between the mitotic and apoptotic index (M/A). *P < 0.05 between the groups indicated by the bars in (A) and (C), and respect to other groups in (B). The data were analyzed by ANOVA I with subsequent analysis of Newman–Keuls.

To study the influence of ovarian steroids on tumor progression, the relationship between the mitotic and apoptotic index was calculated dividing the mitotic figures by the apoptotic bodies for each tumor. We observed that treatment with only E2 significantly increased mitosis in tumors compared to the group treated with V (Fig. 3A; *P* < 0.05). Regarding apoptosis, the combined treatment with E2 + P4 significantly increased (Fig. 3B; *P* < 0.05) the number of apoptotic bodies with respect to the other groups. The relation between the mitotic and apoptotic index decreased in the E2 + P4 group compared to E2 alone (Fig. 3C). Therefore, treatment with only E2 would induce proliferation whereas E2 + P4 would promote apoptosis in colon tumors.

### Treatment with E2 + P4 decreases cell proliferation in colon tumors

**Figure 4:**
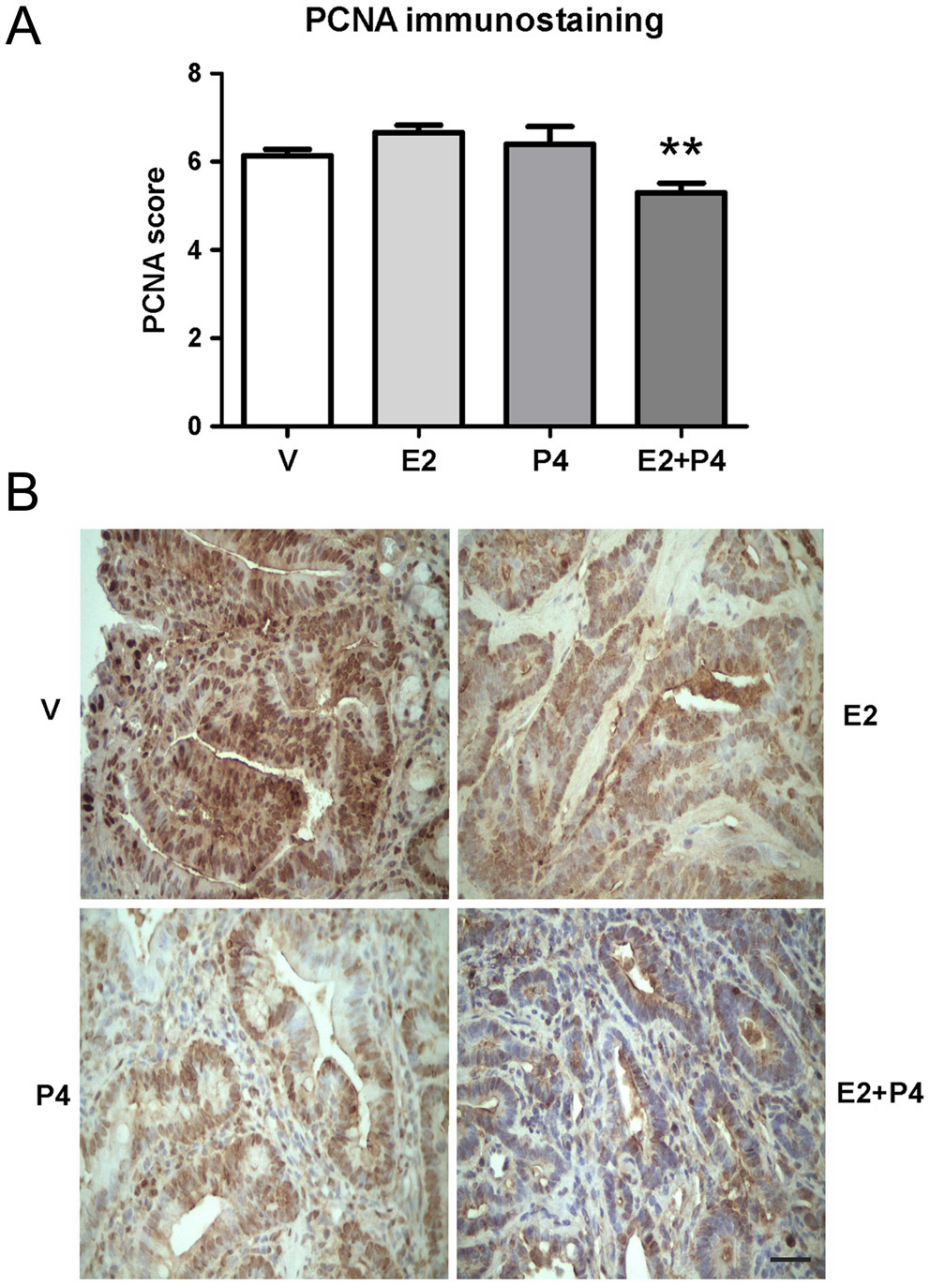
Expression of PCNA in colon tumors from Sprague–Dawley rats. The expression of this proliferation marker was evaluated by immunohistochemistry. (A) The score of immunostaining was calculated adding the percentage (0 = 0%; 1 ≤ 10%; 2 = 10–33%; 3 = 34–65%, 4 ≥ 66%) of tumor cells stained and the intensity (1 = weak, 2 = moderate, 3 = severe) of expression. (B) Representative microphotographs (400×) of PCNA immunostaining in colon tumors from rats treated with V, E2, P4 and E2 + P4. Black scale bar represents 50 μm. **P < 0.01 compared to the other groups. The scores were analyzed by the Kruskal–Wallis and Dunn’s test.

To study further the effect of ovarian steroids on tumor cell proliferation, we analyzed the expression of PCNA by immunohistochemistry (IHC). We observed that the tumors from group E2 + P4 showed a lower expression of PCNA compared to the other groups (Fig. 4; *P* < 0.01), indicating an antiproliferative effect when both hormones are present.

### E2 + P4 treatment increases cell apoptosis in colon tumors

**Figure 5:**
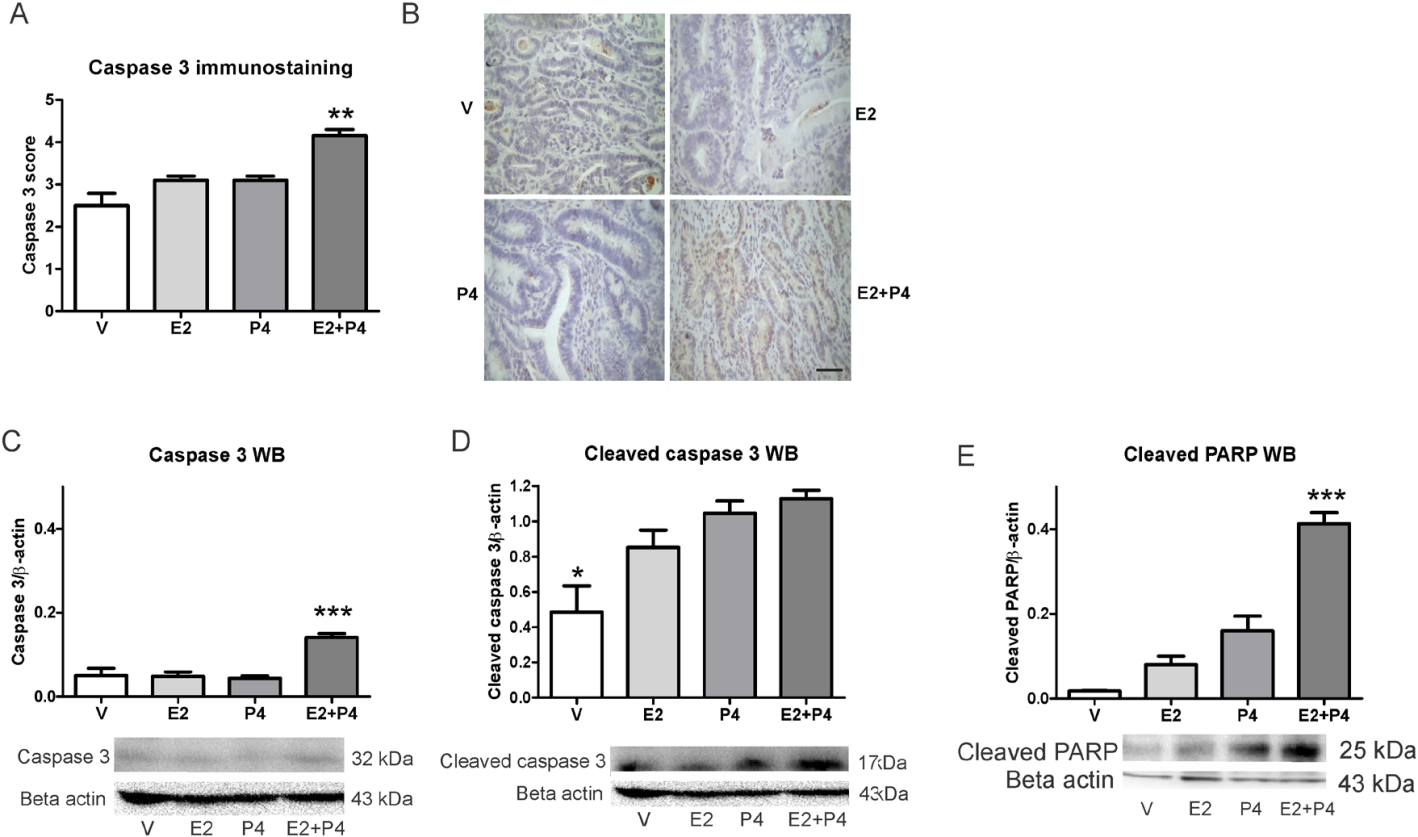
Expression of caspase 3 and cleaved PARP in colon tumors from Sprague–Dawley rats. Total caspase 3 expression was measured by IHC and the cleaved form was determined by WB. (A) The score of immunostaining was calculated adding the percentage (0 = 0%; 1 ≤ 10%; 2 = 10–33%; 3 = 34–65%, 4 ≥ 66%) of tumor cells stained and the intensity (1 = weak, 2 = moderate, 3 = severe) of expression. (B) Representative microphotographs (400×) of total caspase 3 immunostaining in colon tumors from rats treated with V, E2, P4 and E2 + P4. Black scale bar represents 50 μm. (C) Expression of total caspase 3 in colon tumors evaluated by WB. (D) Expression of cleaved caspase 3 in colon tumors evaluated by WB. (E) Expression of cleaved PARP evaluated by WB. The bands were normalized against beta actin. *P < 0.05, **P < 0.01, ***P < 0.001, compared to the other groups. WB data were analyzed by ANOVA I with subsequent analysis of Newman–Keuls and the immunohistochemical scores were analyzed by the Kruskal–Wallis and Dunn’s test.

Since we observed an increase in cell apoptosis due to the combined treatment of E2 + P4, we analyzed the expression of proteins associated with this form of programmed cell death. Figure 5 shows the expression of caspase 3 and cleaved PARP in tumors from the different groups. The treatment with E2 + P4 increased the expression of total caspase 3 compared to the other groups, demonstrated by IHC and WB (Fig. 5A, B and C, *P* < 0.01 and *P* < 0.001). Tumors from the V group showed a decreased expression of cleaved caspase 3 compared to the other groups (Fig. 5D; *P* < 0.05). We also observed an augmented expression of cleaved PARP in the E2 + P4 group (Fig. 5E; *P* < 0.001). These results confirm the activation of the apoptotic process by the treatment with E2 + P4.

### E2 + P4 treatment induces apoptosis through the extrinsic pathway

**Figure 6:**
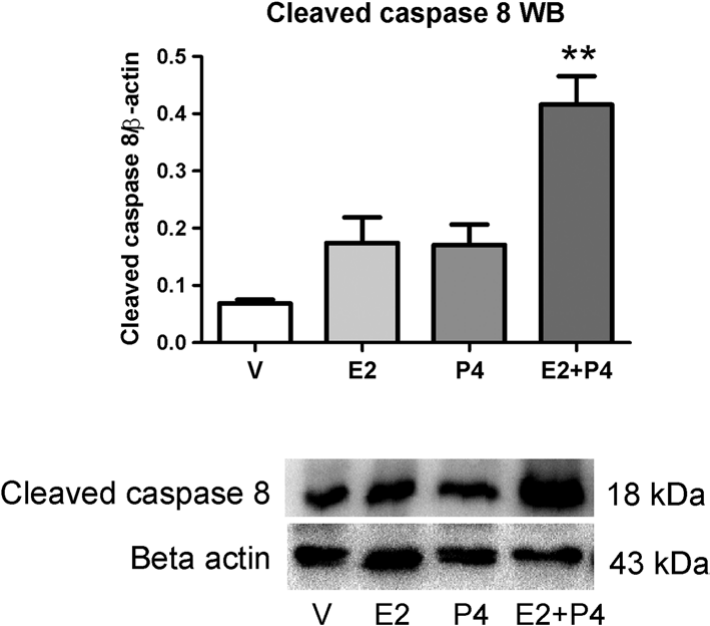
Expression of cleaved caspase 8 in colon tumors from Sprague–Dawley rats. The expression was measured by WB. The bands were normalized against beta actin. **P < 0.01 compared to the other groups. The data were analyzed by ANOVA I with subsequent analysis of Newman–Keuls.

To elucidate the pathway involved in the activation of apoptosis in the tumors from the E2 + P4 group, we analyzed the proteins of the BCL2 family and caspases 8 and 9. No differences were observed in the expression of BAX, BCL2 and cleaved caspase 9 (data not shown), suggesting that the intrinsic pathway is not involved in the apoptosis produced by E2 + P4 in colon tumors. Therefore, the expression of cleaved caspase 8 was analyzed by WB to determine if the apoptosis observed was driven by the extrinsic pathway. The results showed a significant increase (Fig. 6; *P* < 0.01) in the expression of cleaved caspase 8 in tumors from E2 + P4 compared to the other groups. Therefore, the extrinsic pathway may be involved in the apoptotic process produced by the treatment with E2 + P4 in colon tumors.

### E2 + P4 treatment increases the expression of ERB

**Figure 7:**
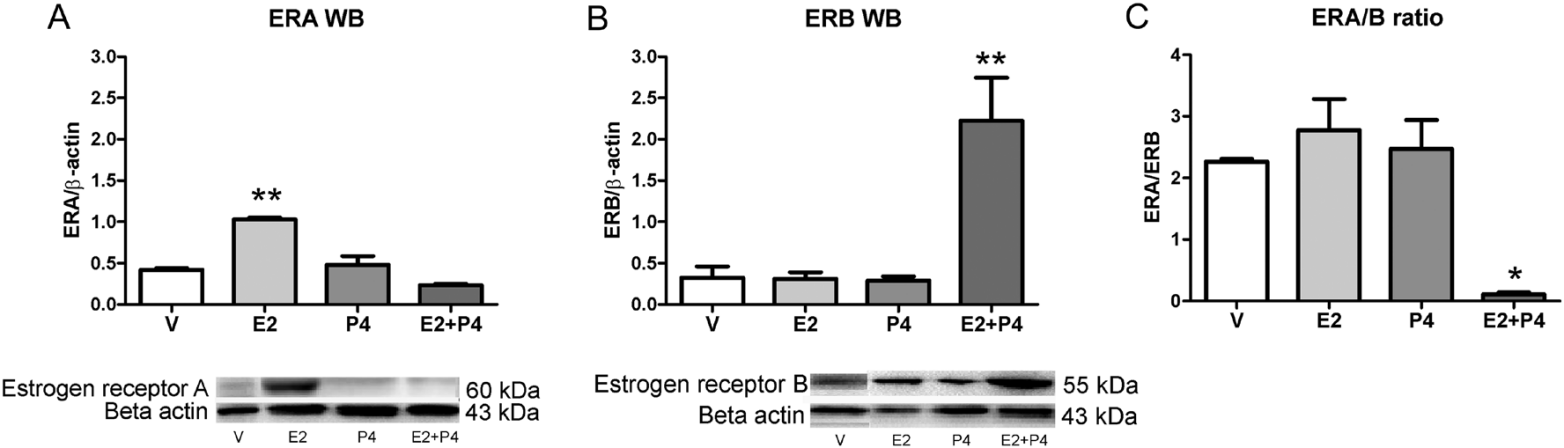
Expression of estrogen receptor isoforms in colon tumors from Sprague–Dawley rats. The A (A) and B (B) isoforms from ER were evaluated by WB. The bands were normalized against beta actin. (C) Ratio between the expression of A and B isoforms of ER in each tumor. *P < 0.05 and **P < 0.01 compared to the other groups. The data were analyzed by ANOVA I with subsequent analysis of Newman–Keuls.

To analyze the status of hormone receptors responsible for the action of the ovarian steroids, the expressions of ERA and ERB were quantified by IHC and WB. IHC did not show differences in the expression and localization of both receptors between the different treatments (data not shown). By WB, we observed an increase in ERA expression in the E2 group compared to the other groups (Fig. 7A; *P* < 0.01). As for the ERB, we observed a consistent increase in the E2 + P4 group compared to the other groups (Fig. 7B; *P* < 0.01). When we analyzed the ratio between the two receptor isoforms, a lower ratio (Fig. 7C; *P* < 0.05) was observed for tumors from combined treatment compared to the other groups.

### Treatment with E2 induces the expression of PR

**Figure 8:**
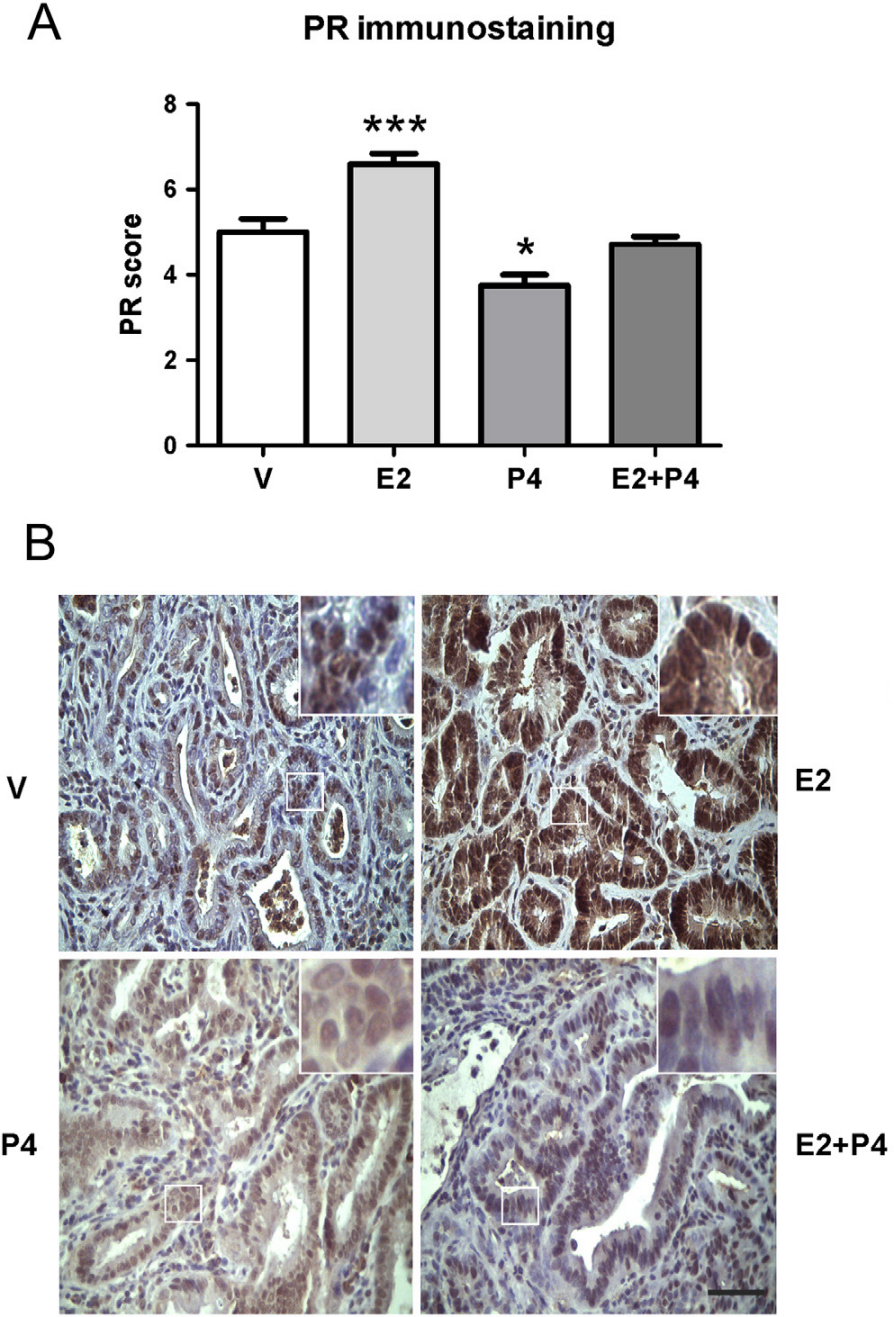
Expression of progesterone receptor in colon tumors from Sprague–Dawley rats. The expression of PR was measured by IHC. (A) The score of immunostaining was calculated adding the percentage (0 = 0%; 1 ≤ 10%; 2 = 10–33%; 3 = 34–65%, 4 ≥ 66% of tumor cells stained) and the intensity (1 = weak, 2 = moderate, 3 = severe) of expression. (B) Representative microphotographs (400×) of PR immunostaining in colon tumors from rats treated with V, E2, P4 and E2 + P4. Black scale bar represents 50 μm. An insert has been included for better cell localization display. *P < 0.05 and ***P < 0.001 compared to the other groups. The data were analyzed by the Kruskal–Wallis and Dunn’s test.

Since one of the most conspicuous effects of E2 is to induce the expression of the PR (24, 25), we analyzed by IHC PR expression to assess for the action of E2 on the tumors. On the one hand, we observed that the treatment with E2 increased PR score compared to the other groups (Fig. 8; *P* < 0.001). On the other hand, treatment with P4 decreased its expression compared to all groups (*P* < 0.05). The receptor was located both in cytoplasm and nucleus in all treatments. No differences were observed between the groups in the expression of the receptor isoforms determined by WB (data not shown).

### *ESR1* expression correlates with *ESR2* and *PGR* expressions in human colon cancer

**Figure 9:**
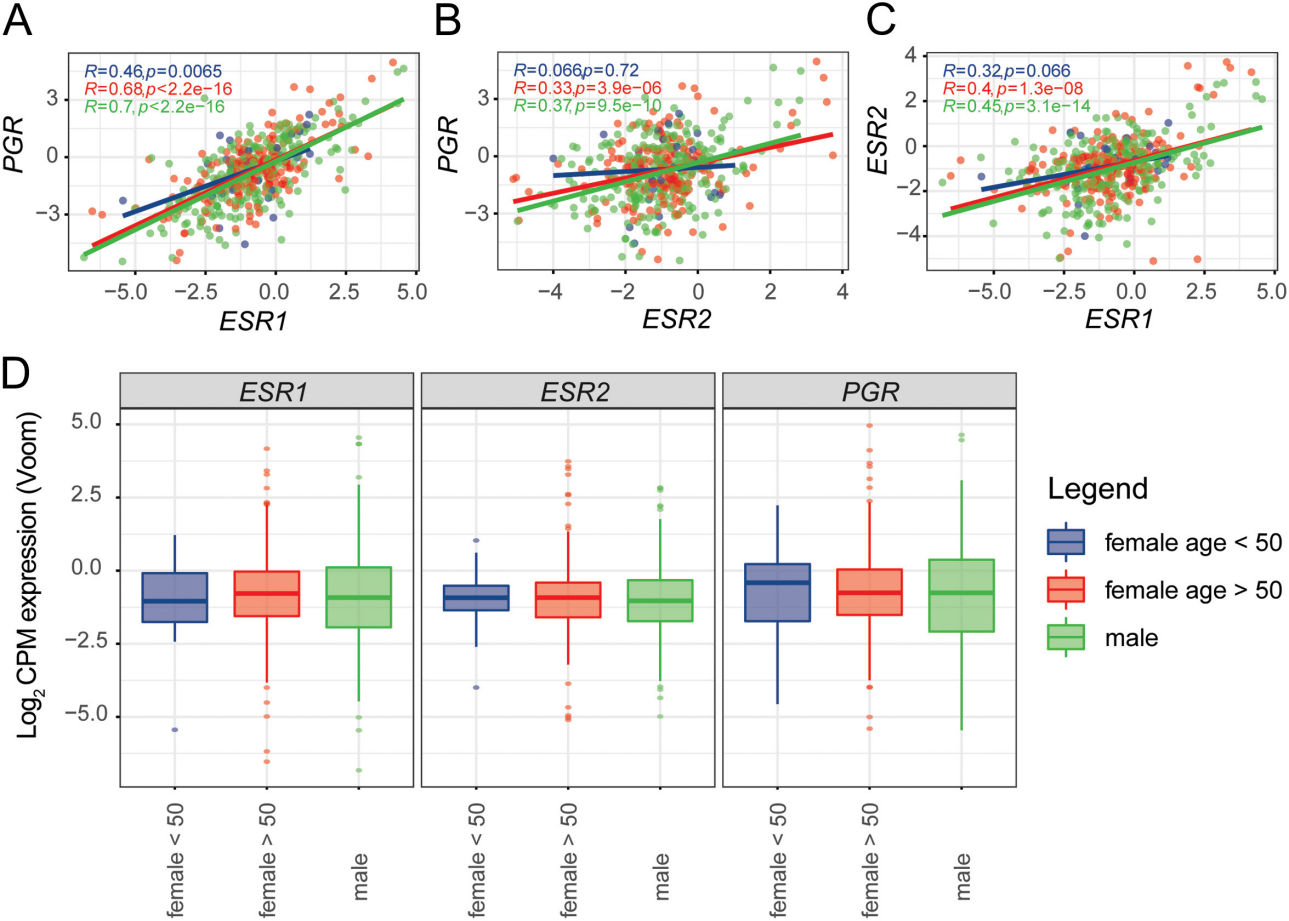
*ESR1*, *ESR2* and *PGR* expression levels in colon tumors from the TCGA COAD cohort. (A) Correlation between *ESR1* and *PGR* expressions. (B) Correlation between *ESR2* and *PGR* expressions. (C) Correlation between *ESR1* and *ESR2* expressions. (D) Levels of expression of *ESR1*, *ESR2* and *PGR* in colon tumors. Transformed gene expression correlation between the three genes was evaluated using Pearson's correlation coefficient with its corresponding *P* value. The direction of the relation was calculated using simple linear regression and depicted as a straight line with a slope in a scatterplot.

To compare the results obtained in our animal model with human colon tumors, we performed a gene expression analysis including all colon cancers from the TCGA COAD cohort. We discriminated tumors from men (M) and women over 50 years old (postM, considered postmenopausal women), and women under 50 years old (preM representing premenopausal women). We did not find any change in the levels of expression of the three steroid receptor genes between the groups. However, we found a strong correlation between the expression of *ESR1* and *PGR* in preM (*P* < 0.01) and in M and postM (*P* < 2.2 × 10^−16^) (Fig. 9A). Moreover, *ESR2* expression correlated with *PGR* expression in both M and postM groups (*P* < 4 × 10^−6^) (Fig. 9B). Additionally, a significant correlation between *ESR1* and *ESR2* was found in the M and postM groups (*P* < 2 × 10^−8^) (Fig. 9C).

## Discussion

In the present study we used the carcinogen DMH to develop the experimental model of colon cancer. This drug has been widely used to induce adenocarcinoma of colon and rectum in rodents with high incidence and specificity (19, 26, 27). The histopathology of tumors developed with this carcinogen is similar to that observed for sporadic colon tumors in humans (28, 29) and is a highly versatile model for studies of chemoprevention, genetics and biology of colon cancer (30). Coincidentally, the tumors developed in our study were similar to those observed in human carcinogenesis. At the same time, the treatment with DMH produced a high tumor incidence in all groups. The lack of differences in incidence may be due to the potent effect of DMH which could not be reversed by any of our hormonal treatments. In human epidemiological studies, ovarian steroids have a protective effect on CRC, which varies between 20 and 40% (31, 32). However, women who had received the therapy when they were diagnosed with CRC presented a more advanced stage in the disease. Therefore, although the estrogens are initially protective, once the CRC has been developed exogenous estrogens increase their growth (4). In a meta-analysis carried out with data published up to 2010, the authors concluded that only a few studies had examined the associations between estrogen therapy vs combined therapy (estrogens plus progestins) and CRC (33). They also concluded that the use of combined therapy significantly reduced the risk of CRC, while the use of only estrogen produces more variable effects. The evidence of a possible differential risk associated with cyclic vs continuous combination therapy or depending on the administration routes is scarce (34). Some *in vivo* studies have indicated that estrogens inhibit the proliferation of CRC (5), while others suggest that they cause mitogenic effects (4). These controversies may be due to the differences in the experimental models in terms of initiation of therapy (before or after carcinogenic induction), types and doses of the steroids used, duration of the experiment, among others. In addition, our criterion to kill the animals was the appearance of symptoms compatible with the presence of tumors, such as the presence of diarrhea or weight loss. The latency of the appearance of symptoms was shorter after treatment with P4 compared to E2 + P4. That result might be due to a more evident symptomatology of the animals more than to a shortening of latency of tumor development. In fact, the location of the tumors was mainly in the distal colon of rats treated with P4 alone, different to the other groups. Besides, we found no significant difference in multiplicity. This observation may be due to the limitations of the animal model, the carcinogen used and the time of the killing.

Additionally, the mitotic index was higher in tumors from the E2 group compared to the V group. This effect was reversed by the combined treatment with P4. When we analyzed the apoptotic index, we found a decrease by the E2 + P4 treatment compared to the other groups. The augmented mitosis induced by E2 alone and the diminished apoptosis due to the combined treatment makes the mitotic and apoptotic indices ratio be significantly lower after E2 + P4 treatment. This observation was confirmed by a reduction in the expression of PCNA and an increased expression of total caspase 3 and its cleaved form. In addition, the E2 + P4 treatment augmented the expression of cleaved PARP and cleaved caspase 8, but did not modify the expression of BAX, BCL2 and caspase 9. Taken together all these results indicate that the apoptotic effect produced by the combined treatment of E2 + P4 on tumors is mainly driven through the extrinsic pathway. These results are in concordance with previous studies describing that the effects produced by P4 are opposite to those of E2 (18). In addition, there are also studies showing that estrogen-only hormone replacement therapy does not produce changes in the prevalence or survival after developing colon cancer in women. The authors postulate that P4 is necessary for protection against this type of cancer because of the modulation that exerts on the effects of estrogens on carcinogenesis (16).

On the other hand, there are controversial results regarding the expression of ERA in the colon (35, 36). Using IHC no expression of ERA in samples from patients with CRC has been reported (37, 38). However, other studies show the expression of ERA in CRC, but at very low levels compared to the B isoform (3, 16). This controversy may be due to changes in the way samples were processed, the method or antibodies used, or the level of staining to consider a sample positive (39). In the present study, we observed an increase in the expression of ERA by the treatment with E2 and a decrease by E2 + P4 administration. The higher expression of ERA produced in tumors by E2 can explain the effects observed on proliferation and the mitotic and apoptotic index. The activation of ERA is known to activate proliferation in epithelial cells, thus promoting carcinogenesis (40). Also, E2 can activate the G protein-coupled estrogen receptor (GPER), which has been described to increase proliferation in CRC (41, 42). We observed that the treatment with P4 reduces ERA expression and this effect is enhanced when both hormones are present. Recently, Mohammed *et al*. reported that P4 promotes direct interaction between its receptor and the ERA in breast cancer, which redirects the transcriptional activity of ERA, blocking proliferative actions caused by E2 alone (43). This should be one of the mechanisms involved in the decrease of the mitotic and apoptotic index induced by P4 in rats receiving also E2. Still, previous studies have postulated the loss of ERB in colon cancer progression with an increase in the expression of ERA, which would relate this receptor with a more invasive profile (44). Another study has reported that soy and estrone protect mice from the development of colon cancer even when they do not express ERA, suggesting that this isoform is not necessary to mediate the protective effects of estrogens in colon cancer (45). Therefore, although there are several reports on the presence of ERA in the colon, the signaling of estrogens is mediated predominantly through the ERB (46). We observed a low expression of ERB in tumors from groups treated with E2 or P4, while its expression was significantly augmented by the combined treatment of E2 + P4. Consequently, the ratio ERA/ERB was significantly decreased by the combined treatment. The proapoptotic effects observed by the combined treatment are related to the increase of the ERB expression. The antiproliferative and proapoptotic role of ERB in colon tumors have been described, and it is believed that those effects may be due to the combination of several events such as regulation of the cell cycle, decrease in the expression of oncogenes such as *MYC* and *MYB*, regulation of the anti-inflammatory response and an increase in DNA repair capacity (47). The loss of ERB expression in the normal colon produces a greater risk of suffering cancer and also, once the disease has developed, a lower expression is associated with a poor survival in patients (13, 48).

Finally, regarding the PRs, there are some studies that report the absence of PR expression in colon tumors and no effect of progestins on carcinogenesis in animal models (15). However, other studies detect the implication of P4, where the expression of PR increases in the order of normal colon-adenoma-adenocarcinoma, demonstrating a role for this receptor in the disease (16). In the present study we observed a high expression of PR in colon tumors due to treatment with E2 and a low expression in the group treated with P4 only. No changes were observed when the A and B isoforms were quantified separately. From these results we conclude that the PR seems to be regulated negatively by its ligand, unlike estrogen receptors that show both self-induced as self-repression. In uterine cells, ERs bound to E2 increase the expression of PR. It seems that in colon tumors this mechanism of regulation remains unchanged. Most of the literature on P4 or progesterone-like compounds is contradictory because the effects of synthetic progestins are different than those of natural progesterone. The difference in chemical structure is profound and results in different actions at the cell level (49). That may account for the controversy in the bibliography, which makes difficult to compare results from distinct studies. Thus, different progestins can be associated with different types of estrogens and different administration regimes (50).

In order to compare the results obtained in our animal model with human colon tumors, we performed a gene expression analysis search including all colon cancers from the TCGA COAD cohort. We can conclude that there is a strong correlation between the expression of *ESR1*, *ESR2* and *PGR*. In the case of *ESR2*, the correlation with *PGR* is not statistically significant in the group of premenopausal women. We would expect to see a positive correlation in this particular case, since in our animal model the presence of ovarian steroids produces an increase in the ERB at protein level. Since these results are obtained measuring the mRNA levels, the expression of proteins could be changed due to posttranscriptional regulation. Our results from the TCGA COAD cohort suggest a role for ovarian hormone receptors in human colon carcinogenesis.

In conclusion, we demonstrated that the presence of natural ovarian steroids is necessary to observe protective effects on colon cancer. Consequently, to study the effect of P4 on the development of colon cancer is necessary since it is a hormone present in the early stages of the disease in women at childbearing age. Moreover, the number of postmenopausal women using natural progesterone instead of synthetic progestins in hormone replacement therapy is increasing. Therefore, the mechanism of action by which both hormones, E2 and P4, contribute to the proapoptotic effect observed in colon tumors remains to be elucidated.

## Declaration of interest

The authors declare that there is no conflict of interest that could be perceived as prejudicing the impartiality of the research reported.

## Funding

This work was partially supported by grants from Instituto Nacional del Cáncer (Ministerio de Salud, Argentina) and from CONICET (Consejo Nacional de Investigaciones Científicas y Técnicas, Argentina).

## Author contribution statement

C V S participated in the design, performed the experiments and drafted the manuscript. F E S and F C V A performed part of the experiments and helped draft the manuscript. L E Z contributed to the discussion of the results. S N S performed the histopathological analysis of the samples. M E G-G performed the gene expression analysis. V P C contributed to the discussion of the results and drafting the manuscript. C M L F performed part of the experiments and drafted the manuscript. R W C participated in its design, performed part of experiments and helped draft the manuscript. All authors read and approved the final manuscript.
